# Long-term results and comparison of the three different high tibial osteotomy and fixation techniques in medial compartment arthrosis

**DOI:** 10.1186/s13018-017-0547-6

**Published:** 2017-03-16

**Authors:** Gökhan Polat, Halil İbrahim Balcı, Mehmet Fevzi Çakmak, Mehmet Demirel, Cengiz Şen, Mehmet Aşık

**Affiliations:** Department of Orthopaedics and Traumatology, Istanbul University Istanbul Medical Faculty, 34093 Çapa-Fatih/Istanbul, Turkey

**Keywords:** High tibial osteotomy, Closing wedge, Opening wedge, External fixator, Medial compartment arthritis, Long-term comparison

## Abstract

**Background:**

The purpose of this study is to report and analyze the long-term outcomes of the patients who underwent high tibial osteotomy (HTO) with three different techniques for the treatment of medial compartment arthrosis.

**Methods:**

A total of 187 patients (195 knees) who underwent HTO between 1990 and 2010 were retrospectively evaluated. Eighty-eight knees, opening-wedge osteotomy with Puddu plate (group A); 51 knees, transverse osteotomy below the tubercle with external fixator (group B); and 29 knees, closing-wedge osteotomy with staple fixation (group C) were included in the study. The patients (mean age 44.9 ± 10.6 years, mean follow-up of 12.4 ± 3.2 years) were called for final controls and survival rates of the knees, and functional evaluations of the patients were performed using Knee Society Score (KSS) and Hospital for Special Surgery (HSS) knee score assessments.

**Results:**

In the comparison of the three groups, there were no differences regarding the mean age, preoperative arthrosis levels, or preoperative deformity analyses (n.s.). The main finding of these comparisons showed that the closing-wedge osteotomy has the greatest lateralization effect on mechanical axis deviation (MAD) (*p* = 0.024), the greatest valgization effect on medial proximal tibial angles (MPTA) (*p* = 0.026), and the lowest posterior tibial slope (PTS) angles (*p* = 0.032) in comparison to the other groups. There were no functional differences between the three groups in the long-term assessment of patients with KSS and HSS knee scores. According to the Kaplan–Meier survival analysis, the probability of the survival of the native knee joint after HTO was 93.4% in 5 years and 71.2% in 10 years in our study group. During the follow-up of the 168 knees, revision surgery with total knee replacement was needed in 27 knees (16%). The mean time from HTO to total knee replacement was 8.9 years in these patients.

**Conclusions:**

HTO has acceptable long-term clinical and functional results that should not be underestimated by orthopedic surgeons under pressure to perform arthroplasty operations.

## Background

The management of medial compartment arthrosis remains challenging in orthopedic practice, particularly in young active patients [1–3]. High tibial osteotomy (HTO) is the main biologic treatment option for most of these cases, especially in those with an intact lateral compartment [4–6]. Some degree of varus malalignment should be expected in patients with the indication of HTO. The aim of an HTO is to redirect the mechanical axis from the degenerated area of the joint to the relatively well-preserved compartment [4–6].

There are many surgical options for medial compartment arthrosis, such as arthroscopic debridement, HTO, resurfacing procedures, unicompartmental arthroplasty, and total knee arthroplasty [7–10]. Although biologic treatment methods should be chosen primarily in young and active patients, HTO procedures have been ignored by most surgeons due to the technological improvements and the early term success of the resurfacing and unicompartmental knee arthroplasty procedures [11, 12]. HTO is a good option in middle-aged or older patients who are not good candidates for arthroplasty procedures due to the social differentiation and habits of the patients or the reluctance of the patients to undergo arthroplasty [13, 14].

The purpose of this study is to report and analyze the long-term outcomes of the patients who underwent HTO as a treatment for medial compartment osteoarthritis and to evaluate the survival rates of the joints using a comparison of three different techniques.

## Methods

A total of 187 patients (195 knees) who underwent HTO in the same department due to medial compartment arthrosis between 1990 and 2010 were retrospectively evaluated and included in our study. The following patients were excluded from this study: 25 patients (27 knees) who we could not contact, 13 patients who could not complete a final assessment, 2 patients who had a lower extremity injury after surgery, 2 patients who had undergone hip arthroplasty, and 8 patients who did not want to participate in the study.

A total of 162 patients (168 knees) were reached at the last follow-up and were included in the study. The mean age of these patients was 44.9 ± 10.6 years (22–68), with a mean follow-up of 12.4 ± 3.2 years (5–22). Of the 168 knees of the 162 patients, 88 knees had undergone an opening-wedge osteotomy and fixation by Puddu plate (group A), 51 knees had undergone a transverse osteotomy below the tubercle and had been fixated externally (group B), and 29 patients had undergone a closing-wedge osteotomy and fixation by staples (group C).

Preoperative and operative patient data were obtained from operative charts and patient files. The patients were called for final assessments, and the mean follow-up durations were set. Functional evaluations included measurements of range of motion (ROM), Knee Society Score (KSS) and the Hospital for Special Surgery (HSS) knee score. The indication for HTO operation had been accepted as grade 2 or grade 3 medial compartment arthrosis that had been evaluated by using standing AP and lateral x-rays according to the Kellgren-Lawrence classification [15]. Osteotomy types were based on surgeon preferences. Preoperative and postoperative mechanical axis deviation (MAD), posterior tibial slope (PTS), and medial proximal tibial angles (MPTA) were measured via long leg weight-bearing orthoroentgenograms. Subgroup evaluations were performed according to the osteotomy techniques to compare functional results and survival rates of the knees.

All patients signed an informed consent form to participate in the study. The local ethical committee approved this study.

### Surgical techniques and postoperative rehabilitation

Detailed information on surgical interventions was provided to all patients. All patients signed an informed consent form that detailed the operative technique to be performed. Patients were also educated about the rehabilitation program.

#### Opening-wedge osteotomy

Under tourniquet, an approximately 8–10 cm incision was made parallel to the anterior border of the medial collateral ligament adjacent to the anteromedial aspect of the proximal tibia. The medial collateral ligament is stripped from the tibia posteriorly to expose the whole proximal medial surface of tibia. Then, under the guidance of the fluoroscopy, a guide wire was advanced medially from 1–2 cm distal to the level of the joint up to the lateral cortex, and an osteotomy apparatus was mounted on the guide wire. Afterwards, a second Kirschner wire was introduced at an appropriate angle up to the lateral cortex. After that, medial, anterior, and posterior cortices were cut immediately under the guide wire up to 1 cm to the lateral cortex. The osteotomy was opened till the lower limb mechanical axis passed across the Fujisawa point [16]. The osteotomy was fixed with a Puddu plate (Arthrex, Naples, Florida), and the osteotomy site was grafted with an autogenic or allogenic bone graft. After the closure of the layers and the placement of a drain, the patient’s knee was placed into a hinged immobilizer. The patients were allowed to walk via two crutches without full weight bearing for 6 weeks. Full weight bearing was allowed 6 weeks postoperatively, and strengthening exercises were initiated.

#### External fixator

The fibular osteotomy was performed prior to the application of the frame in the middle thirds of fibula. A previously prepared frame that was composed of three carbon rings of a circular external fixator (Tasarım Medical, Turkey) was applied to the extremity. With the guidance of fluoroscopy, the rings were secured to the tibia using 1.8-mm stainless steel wires and 6-mm stainless steel half pins. Using an anteromedial skin incision approximately 4 cm long and starting inferomedial to the tibial tubercle, the tibial osteotomy was performed 1–2 cm distal to the tibial tubercle using the multiple drilling osteotomy technique. An acute intraoperative correction was performed with a medial opening wedge and translation of the distal tibia. The operation ended after checking the MAD with fluoroscopy that passed from the Fujisawa point. Minor corrections in the mechanical axis alignment were performed at the early postoperative follow-up visits after the evaluation of postoperative orthoroentgenograms. Patients were allowed to weight bear as tolerated.

#### Closing-wedge osteotomy

An approximately 8–10-cm curved oblique incision was utilized extending from the tip of the fibula to the tibial tuberosity anteriorly before descending approximately 3 cm along the lateral border of the tibial tuberosity. After the opening of the anterior compartment, the proximal tibia was exposed by a periosteal elevator. A blunt retractor was then placed around the posterior border of the tibia. With caution to the patellar tendon and posterior tibial structures, the desired amount of wedge was resected by a saw blade and osteotome according to the preoperative measurements. After excision of the wedge, the medial cortex was broken gently with a valgus stress, and the osteotomy was fixed with two or three offset staples. The patients were allowed to walk with a long hinged knee brace via two crutches without full weight bearing for 6 weeks. Full weight bearing was allowed 6 weeks postoperatively, and strengthening exercises were initiated.

### Statistical analysis

The Statistical Package for Social Sciences, version 19.0, software (SPSS, Chicago, IL) was used for statistical analysis. The preoperative values were analyzed using analysis of variance and a post hoc Tukey highly significant difference test. The time between HTO and total knee replacement was referred to as “survival of native joint.” Survival rates were calculated using the Kaplan–Meier method. To standardize the values for comparing preoperative and postoperative scores of different patient groups, ANCOVA (analysis of covariance) was used to compare the delta difference between the subgroups. The level of statistical significance was accepted as *p* < 0.05.

## Results

In the preoperative arthrosis evaluation of the patients, 39 knees had grade 2 arthrosis and 129 knees had grade 3 arthrosis. The patient demographics are summarized in Table 1.

**Table 1:** Demographics of the patients

Demographics	
Number of patients (n)	162 (168 knees)
Mean age	44.9 ± 10.6 years (22–68)
Male/female	65/97
Mean follow-up	12.4 ± 3.2 years (5–22)
Left/right	87/81
Mean MPTA, MAD, tibial slope (preoperative)	83.3 ± 3.1°, 28.2 ± 7.5 mm varus, 7.2 ± 4.2°
Mean MPTA, MAD, tibial slope (postoperative)	89.6 ± 5.5°, 3.5 ± 4.9 mm valgus, 7.8 ± 5.2°
Preoperative KSS	56.3 ± 13.4
Last control KSS	70.3 ± 14.9
Preoperative HSS	51.3 ± 9.7
Last control HSS	64.7 ± 13.5
Preoperative arthrosis level	39 knees grade 2 arthrosis 129 knees grade 3 arthrosis
Last control arthrosis Level	5 knees (3%) grade 2 arthrosis 81 knees (48.1%) grade 3 arthrosis 55 knees (32.7%) grade 4 arthrosis
Revision with total knee arthroplasty	27 knees

The mean MPTA of the patients was 83.3 ± 3.1°, the mean MAD of the patients was 28.2 ± 7.5 mm varus alignment, and the mean tibial slope of the patients was 7.2 ± 4.2°. Postoperatively, the mean MPTA, MAD, and tibial slope values of the patients were 89.6 ± 5.5°, 3.5 ± 4.9 mm valgus alignment, and 8.1 ± 5.2°, respectively (*p* = 0.001). In the functional evaluation of the patients, KSS and HSS knee scores were used. The mean preoperative KSS score of the patients was 56.3 ± 13.4, and the mean preoperative HSS score of the patients was 51.3 ± 9.7. At the last assessment of the patients, the mean KSS and HSS scores of the patients were 70.3 ± 14.9 and 64.7 ± 13.5, respectively.

We detected complications in 13 patients (8%), transient peroneal nerve palsy in 1 patient who had been treated with closing-wedge osteotomy, and implant failure in 3 patients who had been treated with opening-wedge osteotomy. Delayed union in 2 patients and nonunion in 1 patient were revised with autogenous bone grafting and an external fixator. Grade 3 pin tract infections in 3 patients were treated with pin extraction, a deep infection in 1 patient was treated with debridement and antibiotic therapy, and deep vein thrombosis occurred in 2 patients.

**Fig. 1:**
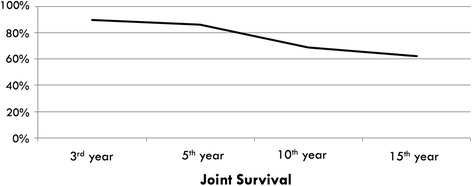
Chart shows the survival analysis of the 168 knees after HTO

During the follow-up of the 168 knees, revision surgery with total knee replacement was needed in 27 knees (16%). The mean time from tibial osteotomy to total knee replacement was 8.9 years in these patients. We uncovered a superficial wound infection in 1 patient and a periprosthetic infection in 1 patient in these revision cases. According to the Kaplan–Meier survival analysis, the probability of the survival of the native knee joint after HTO was 93.4% in 5 years and 71.2% in 10 years in our study group (Fig. 1).

At the last assessment of the patients, an arthrosis evaluation was performed; 55 knees (32.7%) had grade 4 medial compartment arthrosis, 81 knees (48.1%) had grade 3 arthrosis, and 5 knees (3%) had grade 2 arthrosis. Although we had offered total knee arthroplasty to these 49 patients (55 knees) who had grade 4 arthrosis at the last assessment, only 12 of them wanted an arthroplasty operation.

Subgroup analyses were performed according to osteotomy and fixation technique to compare the functional results of patients in the three different groups. Of the 188 knees, 88 knees underwent opening-wedge osteotomy (group A), 51 knees underwent HTO with external fixator (group B), and 29 knees underwent closing-wedge osteotomy (group C). In the comparison of these three groups, there were no differences regarding the mean age, preoperative arthrosis levels, and preoperative deformity analysis (MPTA, MAD, PTS) (n.s.). However, the mean follow-up time in group C was higher than that in the other groups and was statistically significant (*p* = 0.043). In the evaluation of patients’ complications, there were no differences between the three groups. In the comparisons of the mean postoperative correction in MPTA, MAD, and PTA in the three groups, the mean MPTA and MAD were statistically higher in group C in comparison to the other groups (*p* = 0.024, *p* = 0.026, respectively). In addition, the mean postoperative PTS of patients was 8.9 ± 5.1 in group A, 7.8 ± 3.2 in group B, and 6.2 ± 7.3 in group C. These differences were statistically significant (*p* = 0.032). At the last follow-up of the patients, there were no differences between the three groups in the functional assessment according to KSS and HSS knee scores. However, statistically, there were a high number of conversions to total knee arthroplasty in the external fixation group (*p* = 0.035) (Table 2).

**Table 2:** Subgroup comparisons

		Opening wedge (*n* = 88)	External fixator (*n* = 51)	Closing wedge (*n* = 29)	*p* value
Age 44.9 ± 10.6 years		44.6 ± 7.4	45.1 ± 8.5	45.5 ± 9.1	Nonsense
Follow-up (year) 12.4 ± 3.2 years		11.7 ± 5.4	11.3 ± 4.3	13.9 ± 6.2	*p* = 0.043
Mean MPTA	Preop	83.6 ± 3.1	82.5 ± 4.5	83.8 ± 4.3	Nonsense
	Last control	88.7 ± 1.9	89.5 ± 3.1	92.3 ± 6.5	*p* = 0.026
Mean MAD	Preop	28.1 ± 5.5 varus	29.3 ± 4.1 varus	26.4 ± 3.2 varus	Nonsense
	Last control	3.3 ± 3.1 valgus	3.2 ± 2.4 valgus	4.6 ± 6.3 valgus	*p* = 0.024
Mean tibial slope	Preop	7.3 ± 4.5	7.1 ± 3.3	7.1 ± 4.2	Nonsense
	Last control	8.9 ± 5.1	7.8 ± 3.2	6.2 ± 7.3	*p* = 0.032
Complication 13 patients(8%)		7 patients (8.4%) -İmplant failure—3 patient -Delayed union—1 patient -Malunion—1 patient -DVT—1 patient -Deep infection—1 patient	4 patients (8.1%) -Delayed union—1 patient -Grade 3 pin tract infection—3 patient	2 patients (7.1%) -Transient peroneal nerve palsy—1 patient -DVT—1 patient	Nonsense
Revision with TKA 27 patients		12 (13.4%)	10 (19.6%)	5 (17.2%)	*p* = 0.041
KSS	Preop	56.1 ± 15.9	56.2 ± 14.6	57.0 ± 11.7	Nonsense
	Last control	70.4 ± 10.7	70.1 ± 9.8	70.3 ± 12.1	Nonsense
HSS knee score	Preop	51.2 ± 8.9	51.3 ± 7.4	51.5 ± 9.5	Nonsense
	Last control	64.7 ± 10.2	64.5 ± 9.7	65.0 ± 11.2	Nonsense

## Discussion

Although many treatment options have been described for medial compartment arthrosis, HTO is still the most effective method, especially for young to middle-aged patients who are not eligible for arthroplasty procedures [6, 7]. In this retrospective study, we aimed to analyze the long-term outcomes of medial compartment arthrosis patients who were treated with HTO and compared three different osteotomy and fixation techniques.

HTO was popularized by Coventry and Insall in the 1970s, and since that time, many different osteotomy techniques and fixation methods have been described [17–23]. The basic principle of the HTO in medial compartment arthrosis is to redirect the mechanical axis from the degenerated area to the relatively well-reserved lateral compartment [24]. This treatment is a very well-known technique to prolong the lifespan of the native joint in the short- to mid-term follow-up [22, 25]. In the long-term assessment of these patients, the efficacy of the HTO, especially for preserving the native joint, is variable, and survival rates of the native joint range from 61 to 98% in the literature over a 10-year follow-up period [26–29]. In a prospective study, 20 patients were treated with opening-wedge osteotomy and plate fixation, and the authors reported 70% native joint survival with a mean follow-up of 8 years [26]. Polizois et al. reported the results of their closing-wedge osteotomy treatment in 95 patients. The authors reported 61% good-excellent results, with a mean follow-up of 8 years [5, 11, 27]. Gstöttner et al. reported the long-term results of 111 patients (mean age 54, 134 knees) whom they had treated with closing-wedge osteotomy. With a mean follow-up of 12 years (1–25 years), the authors reported 80% native joint survival at 10 years and 66% native joint survival at 15 years [28]. Akuziki et al. reported his series of 132 patients (mean age 63 years) that were treated with closing-wedge osteotomy and plate fixation. With a mean follow-up of 16 years, the authors reported 98% native joint survival at 10 years and 90% native joint survival at 15 years [29]. In our study group, according to the Kaplan–Meier survival analysis, the probability of the survival of the native knee joint after HTO was 93.4% at 5 years, 71.2% at 10 years, and 60.1% at 15 years.

Although conversion from HTO to total knee arthroplasty is technically demanding, especially in cases of previous closing-wedge osteotomies, in the literature, there were no significant differences between these cases and primary total knee arthroplasty cases [30, 31]. In our series of 168 knees, we detected 27 knees in which total knee arthroplasty was needed during the follow-up period. In these 27 knees (16%), the mean time from HTO to total knee replacement was 8.9 ± 3.8 years.

The HTO operation has complications such as neurovascular injury, nonunion, infection, loss of correction, and implant failure. In a recent study, the authors reported a 10.9% complication rate in their patients who were treated with opening-wedge osteotomy [32]. In another study in the literature, the authors compared the complication rates of closing-wedge and opening-wedge osteotomies. The authors reported significantly higher complication rates for nonunion, loss of correction, and material failure in the opening-wedge osteotomy group [33]. We detected complications in 13 patients (8%) in our study. In the subgroup analysis, we found an 8.4% complication rate in group A, an 8.1% complication rate in group B, and a 7.1% complication rate in group C (n.s.). There were no statistically significant differences between the three groups.

In the treatment of medial compartment arthrosis, different types of osteotomies have been described for the valgization of the proximal tibia [17, 23, 24, 26]. Although many studies have investigated the clinical outcomes of these osteotomies, only a few of them compared these osteotomy types regarding biomechanical stability, clinical outcomes, complication rates, etc. [34–36]. The authors compared the external fixation and Coventry wedge technique in 30 patients with a mean follow-up of 28.1 months. The external fixation technique was associated with an outcome comparable to the classic lateral closing-wedge osteotomy [34]. Opening-wedge and closing-wedge osteotomy have been widely used, and some studies have compared these two techniques [37–40]. Hoell et al. reported similar treatment results with these two techniques in a group of 57 patients, with a mean follow-up of 22.5 months [37]. In addition, Brouwer et al. compared these two techniques in his prospective randomized study and reported similar treatment outcomes with a higher implant removal rate in the opening-wedge group [38]. Although the primary result of both osteotomy techniques is the realignment of the mechanical axis to the unaffected lateral compartment, complication rate is still an issue for these osteotomies [6]. Song et al. reported similar complication rates in 194 patients (104 closing wedge and 90 medial opening wedge) who were followed for 12 months and described obesity (body mass index >27.5 kg/m^2^) as an independent risk factor [41]. Some authors investigated this issue in a meta-analysis and reported no significant difference between opening-wedge and closing-wedge techniques according to the analysis of 324 opening-wedge and 324 closing-wedge HTO patients in 20 studies regarding clinical outcomes and complication rates. Although the authors reported similar clinical outcomes in this meta-analysis, they reported a significantly greater posterior tibial slope and a greater angle of correction in the opening-wedge group [39]. Duivenvoorden et al. compared the 6-year outcomes of 92 patients who were treated with opening-wedge and closing-wedge osteotomies. The authors reported higher complication rates in the opening-wedge osteotomy group and a higher rate of conversion to total knee arthroplasty in the closing-wedge osteotomy group with a total conversion rate of 14.1% [40].

Closing-wedge, opening-wedge, and transverse osteotomy below tuberosity with external fixation are three commonly used techniques; in the present study, retrospective subgroup analyses were performed to compare the functional results of patients in these three groups. The main finding of this comparisons showed that the closing-wedge osteotomy has the greatest lateralization effect on MAD, the greatest valgization effect on MPTA, and the lowest PTS angles in comparison to the other groups. There were no functional differences between the three groups in the long-term assessment of patients according to the KSS and HSS knee scores. In the comparison of the three groups regarding revision with total knee arthroplasty, the external fixation group had higher arthroplasty rates after more than 10 years of follow-up (*p* = 0.041). Although the mean preoperative arthrosis level of the subgroups was similar, the body mass index of the external fixation group was higher (*p* = 0.0029). This might be a weakness of our study because arthritic changes are much more common in obese patients [42].

To the best of our knowledge, this is the first study to compare the results of the three techniques in HTO. All patients had a minimum of 5 years of follow-up and a mean follow-up of 12.4 years. Its retrospective nature was the main limitation of our study. Another weakness of the present study was the small sample size and the lack of a satisfaction survey such as SF-12 or SF-36.

## Conclusions

All of the three HTO techniques were effective in the treatment of medial compartment arthrosis, with correction of the MAD and favorable short- to mid-term results. Long-term results of the HTO were hyper-variable: 60 to 90% at more than 10 years of follow-up in studies originating from different cultures [28, 29].

HTO is still the main biological reconstruction method in medial compartment arthrosis with acceptable long-term clinical and functional results that should not be underestimated by orthopedic surgeons who are under pressure to perform arthroplasty operations.

## References

[1] Dennis MG, Di Cesare PE (2003). Surgical management of the middle age arthritic knee. Bull Hosp Jt Dis.

[2] Lützner J, Kasten P, Günther KP, Kirschner S (2009). Surgical options for patients with osteoarthritis of the knee. Nat Rev Rheumatol.

[3] Palumbo BT, Scott RD (2014). Diagnosis and indications for treatment of unicompartmental arthritis. Clin Sports Med.

[4] Ivarsson I, Myrnerts R, Gillquist J (1990). High tibial osteotomy for medial osteoarthritis of the knee. A 5 to 7 and 11 year follow-up. J Bone Joint Surg (Br).

[5] Dowd GS, Somayaji HS, Uthukuri M (2006). High tibial osteotomy for medial compartment osteoarthritis. Knee.

[6] Prodromos CC, Amendola A, Jakob RP (2015). High tibial osteotomy: indications, techniques, and postoperative management. Instr Course Lect.

[7] Feeley BT, Gallo RA, Sherman S, Williams RJ (2010). Management of osteoarthritis of the knee in the active patient. J Am Acad Orthop Surg.

[8] Brouwer RW, Huizinga MR, Duivenvoorden T, van Raaij TM, Verhagen AP, Bierma-Zeinstra SM, Verhaar JA. Osteotomy for treating knee osteoarthritis. Cochrane Database Syst Rev. 2014(12):CD004019. doı: 10.1002/14651858.CD004019.pub4. 10.1002/14651858.CD004019.pub4 PMC7173694 25503775

[9] Hurst JM, Berend KR (2015). Mobile-bearing unicondylar knee arthroplasty: the Oxford experience. Orthop Clin North Am.

[10] Parratte S, Ollivier M, Lunebourg A, Abdel MP, Argenson JN (2015). Long-term results of compartmental arthroplasties of the knee: long term results of partial knee arthroplasty. Bone Joint J.

[11] Labek G, Sekyra K, Pawelka W, Janda W, Stöckl B (2011). Outcome and reproducibility of data concerning the Oxford unicompartmental knee arthroplasty: a structured literature review including arthroplasty registry data. Acta Orthop.

[12] Murray DW, Liddle A, Dodd CA, Pandit H (2015). Unicompartmental knee arthroplasty: is the glass half full or half empty?. Bone Joint J.

[13] Nyland J, Jakob R (2013). Multi-factorial sustainability approach is necessary to preserve knee function following osteoarthritis diagnosis. World J Orthop.

[14] Nyland J, Wera J, Henzman C, Miller T, Jakob R, Caborn DN (2015). Preserving knee function following osteoarthritis diagnosis: a sustainability theory and social ecology clinical commentary. Phys Ther Sport.

[15] Kellgren JH, Lawrence JS (1957). Radiological assessment of osteo-arthritis. Ann Rheum Dis.

[16] Fujisawa Y, Masuhara K, Shiomi S (1979). The effect of high tibial osteotomy on osteoarthritis of the knee: an arthroscopic study of 54 knee joints. Orthop Clin North Am.

[17] Coventry MB (1973). Osteotomy about the knee for degenerative and rheumatoid arthritis: indications, operative techniques and results. J Bone Joint Surg Am.

[18] Insall J, Shoji H, Mayer V (1974). High tibial osteotomy: a five-year evaluation. J Bone Joint Surg Am.

[19] Sangwan SS, Siwach RC, Singh Z, Duhan S (2000). Unicompartmental osteoarthritis of the knee: an innovative osteotomy. Int Orthop.

[20] Schröter S, Gonser CE, Konstantinidis L, Helwig P, Albrecht D (2011). High complication rate after biplanar open wedge high tibial osteotomy stabilized with a new spacer plate (Position HTO plate) without bone substitute. Arthroscopy.

[21] Shiha A, El-Deen MA, Khalifa AR, Kenawey M (2009). Ilizarov gradual correction of genu varum deformity in adults. Acta Orthop Belg.

[22] Staubli AE, De Simoni C, Babst R, Lobenhoffer P (2003). TomoFix: a new LCP-concept for open wedge osteotomy of the medial proximal tibia—early results in 92 cases. Injury.

[23] Chiang H, Hsu H, Jiang C (2006). Dome-shaped high tibial osteotomy: a long-term follow-up study. J Formos Med Assoc.

[24] Lobenhoffer P (2014). Importance of osteotomy around to the knee for medial gonarthritis. Indications, technique and results. Orthopade.

[25] Rudan JF, Simurda MA (1990). High tibial osteotomy. A prospective clinical and roentgenographic review. Clin Orthop Relat Res.

[26] DeMeo PJ, Johnson EM, Chiang PP, Flamm AM, Miller MC (2010). Midterm follow-up of opening-wedge high tibial osteotomy. Am J Sports Med.

[27] Polyzois D, Stavlas P, Polyzois V, Zacharakis N (2006). The oblique high tibial osteotomy technique without bone removal and with rigid blade plate fixation for the treatment of medial osteoarthritis of the varus knee: medium and long-term results. Knee Surg Sports Traumatol Arthrosc.

[28] Gstöttner M, Pedross F, Liebensteiner M, Bach C. Long-term outcome after high tibial osteotomy. Arch Orthop Trauma Surg. 2008;128(1):111–5. doi:10.1007/s00402-007-0438-0. 10.1007/s00402-007-0438-0 17828411

[29] Akizuki S, Shibakawa A, Takizawa T, Yamazaki I, Horiuchi H (2008). The long-term outcome of high tibial osteotomy: a ten- to 20-year follow-up. J Bone Joint Surg (Br).

[30] Karabatsos B, Mahomed NN, Maistrelli GL (2002). Functional outcome of total knee arthroplasty after high tibial osteotomy. Can J Surg.

[31] Kazakos KJ, Chatzipapas C, Verettas D, Galanis V, Xarchas KC, Psillakis I (2008). Mid-term results of total knee arthroplasty after high tibial osteotomy. Arch Orthop Trauma Surg.

[32] Osti M, Gohm A, Schlick B, Benedetto KP (2015). Complication rate following high tibial open-wedge osteotomy with spacer plates for incipient osteoarthritis of the knee with varus malalignment. Knee Surg Sports Traumatol Arthrosc.

[33] van den Bekerom MPJ, Patt TW, Kleinhout MY, van der Vis HM, Albers GHR (2008). Early complications after high tibial osteotomy. J Knee Surg.

[34] Adili A, Bhandari M, Giffin R, Whately C, Kwok DC (2002). Valgus high tibial osteotomy. Comparison between an Ilizarov and a Coventry wedge technique for the treatment of medial compartment osteoarthritis of the knee. Knee Surg Sports Traumatol Arthrosc.

[35] Gaasbeek R, Welsing R, Barink M, Verdonschot N, van Kampen A (2007). The influence of open and closed high tibial osteotomy on dynamic patellar tracking: a biomechanical study. Knee Surg Sports Traumatol Arthrosc.

[36] Magnussen RA, Lustig S, Demey G, Neyret P, Servien E (2011). The effect of medial opening and lateral closing high tibial osteotomy on leg length. Am J Sports Med.

[37] Hoell S, Suttmoeller J, Stoll V, Fuchs S, Gosheger G (2005). The high tibial osteotomy, open versus closed wedge, a comparison of methods in 108 patients. Arch Orthop Trauma Surg.

[38] Brouwer RW, Bierma-Zeinstra SM, van Raaij TM, Verhaar JA (2006). Osteotomy for medial compartment arthritis of the knee using a closing wedge or an opening wedge controlled by a Puddu plate. a one-year randomised, controlled study. J Bone Joint Surg (Br).

[39] Smith TO, Sexton D, Mitchell P, Hing CB (2011). Opening- or closing-wedged high tibial osteotomy: a meta-analysis of clinical and radiological outcomes. Knee.

[40] Duivenvoorden T, Brouwer RW, Baan A, Bos PK, Reijman M, Bierma-Zeinstra SM, Verhaar JA (2014). Comparison of closing-wedge and opening-wedge high tibial osteotomy for medial compartment osteoarthritis of the knee: a randomized controlled trial with a six-year follow-up. J Bone Joint Surg Am.

[41] Song EK, Seon JK, Park SJ, Jeong MS (2010). The complications of high tibial osteotomy: closing- versus opening-wedge methods. J Bone Joint Surg (Br).

[42] Spahn G, Kirschbaum S, Kahl E (2006). Factors that influence high tibial osteotomy results in patients with medial gonarthritis: a score to predict the results. Osteoarthritis Cartilage.

